# The Story of the Insane from Year to Year

**Published:** 1905-10-28

**Authors:** 


### THE STORY OF THE INSANE FROM YEAR TO YEAR.

Seventeenth Annual Series.

### SOMERSET AND BATH ASYLUM, AT WELLS.

At the beginning of the year this asylum contained 860 patients, and at the end of December there were 899 in residence, which is 29 in excess of the number for which proper accommodation exists. There were 163 first admissions, 59 readmissions, 57 discharged recovered, 8 discharged relieved, 24 discharged not improved, and 94 died. The average daily number resident was 864, and the total number under treatment was 1,074. The recoveries stand at 25·6 per cent. of the admissions, and the deaths at 10·7 per cent. of the average number resident. The first of these percentages is decidedly low, and the second is rather higher than the average of county asylums. Of the year's deaths 28 were due to cerebral and spinal diseases, 7 to consumption, 8 to pneumonia, 6 to colitis, and 1 to enteric fever. Of the year's admissions 30 owed their insanity to various moral causes, and among the physical causes intemperance in drink figures 22 times, hereditary influence 51, old age 31, and congenital defect 11. Dr. Laing explains the low recovery rate and somewhat high death rate to the large number of old and feeble cases admitted, and to the very small proportion of recent and acute cases. Of course, these facts would account for a great deal; but Dr. Laing says nothing of the six deaths from colitis, and although the committee refer to two cases of enteric fever, they say that the general health of the patients has been good. During the year 24 nurses passed the examination of the St. John's Ambulance Association, and 8 nurses obtained the certificate of the Medico-Psychological Association; but Dr. Laing does not say whether he has instituted a proper three years' course of instruction for his staff. Charge-attendant Dunton obtained a pension of £46, and this is one of the asylums that has adopted a pension scheme for the staff. Both committee and officials are to be congratulated on this important step, which ought to lead to enthusiasm in the performance of duties and to length of service. The Asylums Committee, "deploring the large increase in the population of the asylums of the county of Somerset and City of Bath, suggest that cases of senile dementia—which form a large proportion—might more generally be treated in workhouses." This is absolutely, true, and if the suggestion were to be acted upon throughout England it would obviate the necessity for that perpetual mess of bricks and mortar at most of our county asylums; but, alas, it never will be acted upon to any appreciable amount unless the capitation grant be withdrawn, or until a similar, if smaller, grant be given to workhouses. Until this is done the Somerset Asylums Committee may as well suggest the advent of the millennium. The weekly cost per head is 8s. 9d.

### WEST RIDING ASYLUM, WAKEFIELD.

Here the numbers were, practically, the same on the last day of the year as on the first when 1,805 patients were under treatment; and there were 52 vacant beds. The first admissions were 443; the readmissions were 103; the recoveries 264; the discharged, relieved, or not improved 48; and the deaths 228. The total number under treatment during the year was 2,351; and the average daily number resident was 1,806. The recoveries calculated on the admissions stand at 48·35 per cent., and the deaths calculated on the average number resident at 12·62, both of these percentages being considerably above the average of county asylums. Of the deaths 74 were caused by cerebral and spinal diseases; 49 by consumption; 20 by pneumonia; 7 by other lung diseases; 4 by enteric fever; and 10 by colitis. Of the year's admissions we learn from Table X. that 159 owed their insanity to domestic trouble, mental anxiety, religion and other moral causes; and that among the physical causes hereditary influence stands first with 175 cases; and intemperance in drink next with 131. As there were 10 deaths from colitis, it is probable that 40 or 50 patients were affected; and Dr. Bevan Lewis' experience leads him to think that "fallacious views have been advanced which are likely to blind our eyes to the true factors of its incidence"; and we are glad that he does not include himself among those who attribute the scourge to bad drainage, overcrowding, impure water, etc., all of which have been eliminated from Wakefield and most other asylums without getting rid of colitis. Nor does "want of cleanliness" as a cause find favour in Dr. Lewis' eyes, and he truly adds "that our large asylums (why not small asylums too?) compare most favourably with the best regulated hospitals in the kingdom." Coming to the cause of the disease he thinks that chronic constipation plays a very important part in its production, and, if we mistake not, he is not the first medical superintendent who has thought so. Certainly Dr. Lewis' experiments in this direction are encouraging enough, but then we know that colitis disappears and reappears in an extraordinary manner; and that, unless chronic constipation were less common in asylums forty years since than it is now, we are without an explanation of the fact that colitis is more common now than it was previously. The question of providing a sanatorium for the West Riding Asylum consumptives has been shelved for the present; and if it should be revived we fully concur that "it would be better to build a cheap and temporary structure for each of the asylums." Nursing and ambulance lectures continue to be given by Dr. Smith to the attendants and nurses, but as yet we hear nothing of a proper three years' course of training. "The Committee wish to express heir appreciation of the management of the institution by Dr. Bevan Lewis." The weekly cost per head is 10s. 7d.

